# Quantifying Postural Control during Exergaming Using Multivariate Whole-Body Movement Data: A Self-Organizing Maps Approach

**DOI:** 10.1371/journal.pone.0134350

**Published:** 2015-07-31

**Authors:** Mike van Diest, Jan Stegenga, Heinrich J. Wörtche, Jos B. T. M Roerdink, Gijsbertus J. Verkerke, Claudine J. C. Lamoth

**Affiliations:** 1 INCAS3, Assen, The Netherlands; 2 University of Groningen, University Medical Center Groningen, Center for Human Movement Sciences, Groningen, The Netherlands; 3 University of Groningen, Johann Bernoulli Institute for Mathematics and Computer Science, Groningen, The Netherlands; 4 University of Groningen, University Medical Center Groningen, Department of Rehabilitation Medicine, Center for Rehabilitation, Groningen, The Netherlands; 5 University of Twente, Department of Biomechanical Engineering, Enschede, The Netherlands; The University of Queensland, AUSTRALIA

## Abstract

**Background:**

Exergames are becoming an increasingly popular tool for training balance ability, thereby preventing falls in older adults. Automatic, real time, assessment of the user’s balance control offers opportunities in terms of providing targeted feedback and dynamically adjusting the gameplay to the individual user, yet algorithms for quantification of balance control remain to be developed. The aim of the present study was to identify movement patterns, and variability therein, of young and older adults playing a custom-made weight-shifting (ice-skating) exergame.

**Methods:**

Twenty older adults and twenty young adults played a weight-shifting exergame under five conditions of varying complexity, while multi-segmental whole-body movement data were captured using Kinect. Movement coordination patterns expressed during gameplay were identified using Self Organizing Maps (SOM), an artificial neural network, and variability in these patterns was quantified by computing Total Trajectory Variability (TTvar). Additionally a k Nearest Neighbor (kNN) classifier was trained to discriminate between young and older adults based on the SOM features.

**Results:**

Results showed that TTvar was significantly higher in older adults than in young adults, when playing the exergame under complex task conditions. The kNN classifier showed a classification accuracy of 65.8%.

**Conclusions:**

Older adults display more variable sway behavior than young adults, when playing the exergame under complex task conditions. The SOM features characterizing movement patterns expressed during exergaming allow for discriminating between young and older adults with limited accuracy. Our findings contribute to the development of algorithms for quantification of balance ability during home-based exergaming for balance training.

## Introduction

Appropriate control of posture underlies many motor skills and is an absolute prerequisite for activities of daily living. Postural control is generally defined as the ability to maintain, achieve, or restore a state of balance during any posture or activity [1]. It is based on the interaction of dynamic sensorimotor processes and by integrating information from various sources such as the vestibular, proprioceptive and visual systems by the central nervous system, which employs adaptive strategies for orientation and balance control. Adequate postural control results in dynamically stable but also highly flexible behavior that alters continuously to accommodate behavioral goals and prevailing circumstances.

Impaired postural control is an important predictor of falls in older adults [2–4]. Deterioration of postural control in older adults develops either due to a specific pathology affecting a particular component of the sensory, motor or central processing systems, and/or as a consequence of a more general age-related deterioration of sensory and neuromuscular control mechanisms [5–7]. It has been shown that deterioration of postural control is characterized by changes in the complexity of movement dynamics as revealed by poor coordination, increased sway variability, loss of predictability and complexity, and decreased stability of sway patterns during standing as well as during dynamic tasks [8–12]. It has been suggested that such changes reflect changes in motor skills and health, that is, the notion that health is characterized by ‘optimal variability’ which reflects the adaptability and flexibility of the underlying control system, while deterioration of postural control is characterized by a loss of this optimal state of variability in motor behavior [13–15], rendering the system more vulnerable to perturbations. In line with this view, increased variability and loss of stability of postural control during standing and walking, as characterized by numerous quantitative metrics (e.g. fractal scaling, sample entropy, recurrent quantification analysis, Lyapunov exponents) have been linked to balance impairments and increased fall risk in the elderly population [16–20].

A rapidly growing number of studies reveal the potential of exergames to train balance in older adults, and reduce fall risk [21]. The concept of exergames is based on generating a virtual world that can be controlled through bodily movements in the physical world, measured with sensors such as the Kinect (Microsoft Corp, Redmond, USA), a commonly available video-game sensor that captures whole-body position data [22,23]. The Kinect is equipped with a video camera and an infra-red depth sensor for motion capture, and generates a colored point cloud of the environment, from which individual body segments are identified [24,25]. The 3D movements of these body segments are captured, thereby allowing the player to control the virtual world. Kinect thus allows capturing real-time whole-body movement data while the user is practicing balance in a complex game task. In a previous study we showed that by using pattern recognition methods, such as Principal Component Analysis, we could accurately extract movement patterns during a weight-shifting ice-skating game, using the Kinect for gameplay and motion capture [22]. The rationale for capturing multi-dimensional position data, i.e. individual body segments, is that weight shifts can be achieved through a wide variety of postural adjustments, or movement patterns. Observing all individual body segments contributing to a weight shift rather than only the weight shift itself, for instance by means of measuring the center of mass, might thus provide additional information about movement patterns expressed during the execution of exergame tasks. Accurate extraction of movement patterns from multi-dimensional Kinect motion capture data is hypothesized to allow quantifying balance ability while the user is performing complex tasks, thereby quantifying and evaluating differences in balance ability due to age and due to task difficulty [22]. It is however not known if balance ability can actually be quantified using whole-body movement data captured during exergaming.

The aim of the present study therefore was to quantify balance ability of young and older adults using multivariate whole-body movement data, captured with Kinect while they are playing an ice-skating exergame under conditions varying in task complexity. To examine the effects of task complexity and age on postural coordination patterns, self-organizing or Kohonen maps (SOM), were applied. SOM is an artificial neural network algorithm that is particularly suitable for organizing complex, high-dimensional data [26], thereby enabling visualization and analysis of human movement patterns in a lower dimension. More specifically, we first identified coordination patterns and variability thereof as a function of task difficulty (movement frequency and amplitude) and age while the user was performing a weight-shifting exergame balance task. We anticipated that the dynamic organization of postural control patterns would be different between young and older persons in terms of the variability of the extracted movement patterns. Secondly, we tested whether the extracted movement patterns, as represented by the SOM features, could be used to train a classifier to discriminate postural control patterns of young adults from that of older adults.

## Methods

### Participants

Twenty older adults (8 women, 12 men; age 71.9 ± 4.0 years) and twenty young adults (11 women, 9 men; age 37.0 ± 16.6 years) participated in this study. Inclusion criteria were as follows: being physically fit, being able to walk without an aid for at least 15 minutes and being 65–85 and 18–60 years of age for older and young adults respectively. Exclusion criteria included: musculoskeletal, visual or neurological impairments, or use of medication that could affect postural control. All subjects provided written informed consent. The research was approved by the Medical Ethical Committee, University Medical Center Groningen (approval number: METc 2013/244), in accordance with the ethical standards of the declaration of Helsinki.

### Procedure and Instrumentation

Subjects played a custom-made ice-skating exergame in which a challenging balance exercise consisting of repeatedly swaying the center of mass in both lateral directions was practiced. This lateral sway movement was chosen because there is substantial evidence that older adults are particularly vulnerable to lateral instability of postural balance and that these age-associated impairments in lateral balance may be an important cause of falls [27]. During gameplay, subjects were instructed to keep their feet on the ground in parallel stance within an 80 x 60 cm^2^ area. The exergame was played in five different conditions: 1) Neutral; sway speed and sway amplitude were self-selected; 2) In-game skating speed was doubled by increasing the game speed parameter by a factor of two; 3) Subjects were instructed to sway at maximum sway frequency with a self-selected sway amplitude; 4) During the sway movement subjects had to lift the leg contra-lateral to the sway direction off the ground; 5) Subjects were instructed to adopt maximum sway amplitude at a self-selected sway frequency. Trials took about 1 minute to complete and were performed twice. Each subject thus performed 10 exergame trials in total. The trial order was randomized, except for trial 1; this was always a ‘condition 1’ trial. The game was controlled using Kinect, which was positioned 2 m in front of the subject at 60 cm height. The game was installed on a laptop computer and displayed on a screen (3.55x2.60 m) 2 m in front of the subject.

Using Kinect and OpenNI SDK v1.5.2.23, 3D positions of 15 body segments including trunk and extremities were obtained at an irregular sample frequency of about 30 Hz. The captured movement data in the young adults were also used for analysis in [22]. The 3D positions of the hands, elbows and feet, as well as all body segment position data recorded in the sagittal plane were discarded, as previous studies showed that these position data are not captured accurately by Kinect, and they are not crucial for the required movement [22,28]. Postures adopted during gameplay were quantified using the nine remaining segmental landmarks, (head, neck, shoulders, lower back, hips and knees). A detailed description of body segment positions can be found in [22].

### Data preprocessing

The position data of the nine body segments, describing the postures adopted during gameplay in the frontal plane, were resampled at 30 Hz using cubic spline interpolation to account for intrinsic sample frequency deviations. For all trials of all subjects the first sway cycle was discarded, and the subsequent ten sway cycles were selected for analysis. A sway cycle was defined as a full left-right-left sway by the shoulders, starting and ending at the outer-most left position of the shoulder segments in the frontal plane. First, the mean sway amplitude and sway frequency were determined for each exergaming trial. Sway amplitude was defined as the mean distance covered by the lower back segment in medio-lateral direction, as measured with Kinect [22]. Sway frequency was defined as the dominant frequency in the power spectrum of the same body segment. Second, the ten sway cycles were time-normalized to 30 frames per sway cycle. Note that phase lag between body segments was preserved. The total number of trials recorded was 400 (40 subjects x 10 trials). Nine (x, y) coordinate pairs described the posture of the subject in a single frame, resulting in an 18-dimensional posture vector ***p***:

$$p=\left(\begin{array}{c} BodySegment1_{x}\\ \vdots \\ BodySegment9_{x}\\ BodySegment1_{y}\\ \vdots \\ BodySegment9_{y} \end{array}\right)$$ (1)

From the original 400 trials 22 trials had to be discarded (8 in the young, 14 in the old), because the number of sway cycles was smaller than ten or because data collection was corrupted, resulting in a total number of 378 trials for analysis. The resulting 378 trials x 300 frames = 113400 posture vectors ***p*** (of dimension 18) were organized in a 3-dimensional array ***P*** with dimensions (*I x J x K*) where *I*, *J* and *K* refer to the total number of trials (378), frames per trial (300), and body segment coordinates (18) respectively. ***P*** consists of elements ***p***_*i*,*j*,*k*_

Data normalization was performed using a modified version of a normalization technique employed by [29]. Their method aims to retain variability between posture vectors created from postural movement, while minimizing the differences between posture vectors caused by anthropometric differences between subjects. In the current study differences in sway amplitude did not only stem from anthropometric differences between subjects, but also from the fact that young adults move closer to the edges of their base of support while swaying than older adults. Instead of dividing centered posture vectors by the mean vector norm, as Federolf *et al*. did, we rescaled centered posture vectors such that all body segments of all subjects have the same mean amplitude in all trials. Note that differences in range of amplitudes between body segments are preserved.

In a first step the mean posture vector $\bar{p_{i}}$ of the K-dimensional posture vectors ***P***_*i*,*j*_ was computed for each trial *i* and subtracted from all posture vectors of the corresponding trial, yielding *J* = 300 *K*-dimensional centered posture vectors ***c***_*i*,*j*_ for each trial *i*:

$$c_{i,j}=p_{i,j}-\bar{p_{i}}=p_{i,j}-\frac{1}{J}\cdot \sum_{j=1}^{J}p_{i,j}$$ (2)

Second, the mean amplitudes of all body segments were computed for each trial and stored in a system of vectors **M** with dimensions (*I x K*), where *I* and *K* refer to the total number of trials (378) and body segments (18), respectively.

$$M=\left(\begin{array}{cccc} m_{1,1} & m_{1,2} & \cdots & m_{1,K}\\ m_{2,1} & m_{2,2} & \cdots & m_{2,K}\\ \vdots & \vdots & \ddots & \vdots \\ m_{I,1} & m_{I,2} & \cdots & m_{I,K} \end{array}\right)$$ (3)

where *m*_*i*,*k*_, the mean amplitude of all *J* = 300 frames of body segment *k* in trial *i*, is given by:

$$m_{i,k}=\frac{1}{J}\cdot \sum_{j=1}^{J}\|c_{i,j}\|$$ (4)

Third, the mean amplitude over all trials, computed for each body segment *k =* 1…18 was given by ${\overset{-}{m}}_{k}$:

$${\bar{m}}_{k}=\frac{1}{I}\cdot \sum_{I=1}^{I}m_{i,k}$$ (5)

Where $\bar{m}$ with elements ${\bar{m}}_{k}$ for *k* = 1…18, has dimensions (18 x 1). In order to rescale the centered posture vectors ***c***_*i*,*j*_ such that all segments in all trials have identical mean values, a final step was taken where a matrix **F** containing scaling factors was computed:

$$F\;=\left(\begin{array}{cccc} {\text{f}}_{1,1} & {\text{f}}_{1,2} & \cdots & {\text{f}}_{1,K}\\ {\text{f}}_{2,1} & {\text{f}}_{2,2} & \cdots & {\text{f}}_{2,K}\\ \vdots & \vdots & \ddots & \vdots \\ {\text{f}}_{I,1} & {\text{f}}_{I,2} & \cdots & {\text{f}}_{I,K} \end{array}\right)$$ (6)

**F** has dimensions (*I* x *K*), where *I* = 378 trials and *K* = 18 body segments.*f*_*i*,*k*_, the scaling factor for all 300 frames of body segment *k* in trial *i*, is given by:

$$\forall_{i}\in (1\ldots I)\;f_{i,k}={\bar{m}}_{k}/m_{i,k}$$ (7)

Each centered posture vector ***c***_*i*,*j*_ was then multiplied by its corresponding scaling factor *f*_*i*,*k*_ yielding 300 normalized 18-dimensional posture vectors ***Ψ***_*i*,*j*_ for each trial, again stored in a 3-dimensional array ***Ψ*** with dimensions (*I* x *J* x *K*). ***Ψ*** consists of elements *Ψ*_*i*,*j*,*k*_ where *Ψ*_*i*,*j*,*k*_ the centered, normalized body segment position is defined as:

$$\forall_{j}\in (1\ldots J)\;\;\psi_{i,j,k}=c_{i,j,k}\cdot f_{i,k}$$ (8)

The latter operation is a simple multiplication of each centered posture vector with the corresponding scaling factor. The effect of this normalization procedure thus is that the average amplitude of each body segment is equal for each trial, and thus for each person, but the differences in movement range of individual segments remain. For example, the mean amplitude of the vertical movements of the spine thus remains different from the mean amplitude of the horizontal movement of the shoulder. The centered and normalized posture vectors ***Ψ***_*i*,*j*_ were concatenated and assembled into one input array with dimensions ((*I* x *J)* x *K) =* 113400 x 18 and used for training a self-organizing map.

### Self-organizing maps

The current study aims to identify movement patterns and variability therein in high-dimensional whole body movement data of young and older adults, without providing *a priori* information about age-related changes in postural control, as well as to classify movement patterns as belonging to either young or older adults. This goal can be generalized to unsupervised recognition of patterns in high-dimensional datasets, and for this purpose artificial neural networks (ANNs) are particularly useful [30]. In the present study we applied a specific type of ANN; the self-organizing map (SOM) or Kohonen map, to compare and visualize coordination patterns in multi-dimensional data sets. SOM is a data visualization algorithm that reduces high-dimensional data to a map consisting of only one or two dimensions, while at the same time displaying similarities in the input data by grouping similar data items together on the map. Input data, in this case the posture vectors, are presented to the SOM hundreds or thousands of times and after each iteration the organization of the map changes such that it displays similarities in the data structures more clearly. A SOM thus allows for learning underlying non-linear patterns in high-dimensional datasets, while compressing and organizing the information to a low-dimensional mapping [26]. SOMs have been used for analysis and classification of movement patterns during various activities including discus throwing [31], walking [32], and cross-country skiing [33]. In the current study the SOM algorithm was used to organize posture vectors, representing states of coordination, on a 2D map.

The working mechanism of a SOM can be described in a few rules. A SOM transforms high-dimensional data, the input vectors, into a low-dimensional discrete map of output nodes. In the current study, an input matrix ***Ψ*** with dimensions ((*I* x *J) x K) =* (113400 x 18) containing all centered normalized posture vectors of all trials was used; that is 113400 input vectors, each with a dimensionality of 18. The output nodes, which define the map, are organized in a 25 x 25 square lattice. Each of the 25x25 = 625 output nodes has an associated weight vector, of which the dimensionality is equal to that of the posture vectors, which is 18. During initialization the output nodes have random weights, therefore the map containing the output nodes can be viewed as a grid with 625 random posture vectors. The SOM working principle is based on competitive learning. In an iterative process each 18-dimensional input vector ***Ψ***_*i*,*j*_ is presented to the SOM. First, the Euclidian distances between the input vector and all output nodes are computed and the output node with the shortest distance to an input vector is declared the best matching unit (BMU). In a second step, the weight vector of the BMU and the output nodes in the proximity of the BMU are updated to match the input vector more closely. Given weight vector *w*_*x*,*y*_(*t*) of output node (x,*y)* at iteration *t*, the updated weight vector *w*_*x*,*y*_(*t+1*) is defined by [26]:

$$w_{x,y}(t+1)=w_{x,y}(t)+h_{x,y}(t)\eta (t)(\psi_{i,j}-w_{x,y}(t))$$ (9)

where *h*_*x*,*y*_(*t*) is the neighborhood function describing the area around the BMU that is updated, which decreases with increasing *t*. *η*(*t*)is the learning parameter, which also shrinks with increasing *t*. For further details concerning the mathematical formulas see [26]. The effect of this update rule is that as more and more posture vectors are presented, the topology of the SOM changes such that similar posture vectors are grouped together on the SOM. When all posture vectors ***Ψ***_*i*,*j*_ have been presented, a second iteration starts, where all posture vectors again are presented to the updated SOM. The effect of the BMU on its neighboring output nodes decreases in each iteration, as specified by *h*_*x*,*y*_(*t*), resulting in fine-tuning of the SOM after a large number of iterations. Starting from an initial state of disorder, the SOM thus gradually shapes into an organized representation where all similar posture vectors are grouped together. In this study, default values from the Matlab 2013b SOM toolbox were used for the neighborhood function and learning-rate parameter. The number of iterations was set to 1000.

### Movement variability

**Fig 1:**
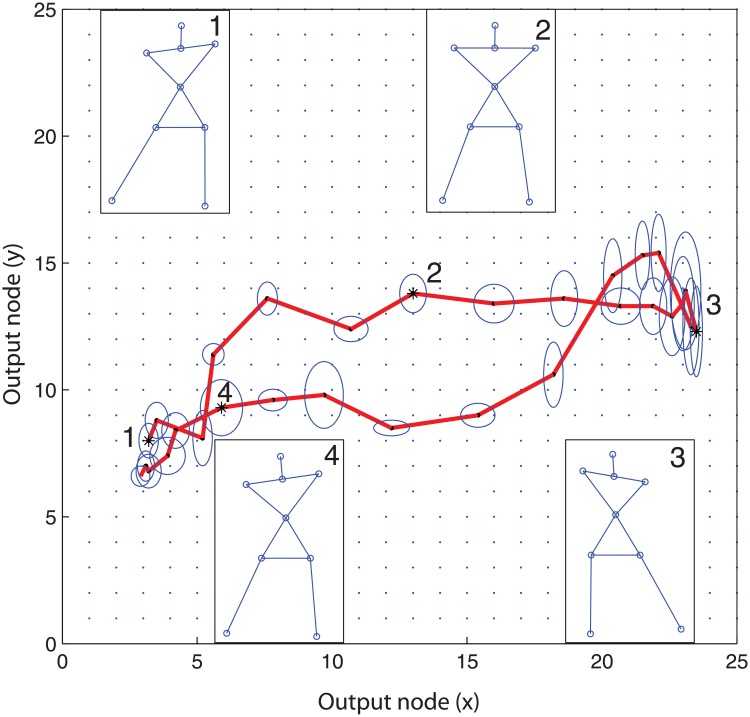
Average sway trajectory of a single subject on the trained SOM. The Best Matching Units (BMUs) of input posture vectors subsequently adopted during an exergame trial form a trajectory of BMUs on the output lattice of a SOM which is trained with movement data of all test subjects. The average sway trajectory of ten sway movements is shown for one older adult playing the ‘maximum sway amplitude’-condition. Blue circles indicate the standard error of the mean for each BMU position. Stick figures are shown for illustration purposes and indicate the average adopted postures 1, 2, 3 and 4 captured at 1%, 25%, 50% and 80% of the time of the sway movement, respectively.

To test the hypothesis that the dynamic organization of postural control patterns would be qualitatively different between young and older persons in terms of the variability of the extracted movement patterns, these patterns were visualized using SOM. Due to the self-organizing nature of the SOM, BMUs of similar input posture vectors ***Ψ***_*i*,*j*_, representing states of coordination, are grouped together on the lattice of output nodes. Moreover, states of coordination subsequently adopted during an exergame trial form a trajectory of BMUs with a smooth structure on the output lattice as shown in Fig 1. This movement pattern, identified in one trial consisting of ten sway cycles, comprises 300 BMUs. To quantify variability in the movement pattern identified from one exergame trial, the variability in the trajectories formed by connecting subsequent BMUs, was computed using a method proposed by Lamb et al. [33]. They computed ‘total trajectory variability’ (TTvar) in three steps. First, the Euclidian distance *d*_*E*_ between al ten sway cycles compared at each BMU *b* was computed. For example, let matrices A and B represent two K-dimensional trajectories of length *s* = J/10, that is 30 frames per sway cycle. The Euclidian distance compared at node *b, $d_{E_{AbBb}}$*, is then given by:

$$d_{E_{AbBb}}=\sqrt{\sum_{K=1}^{K}{\left(A_{bk}-B_{bk}\right)}^{2}}$$ (10)

Second, ${\bar{d}}_{E_{y}}$, the average Euclidian distance between all ten sway cycles in BMU *b*, was computed for each BMU *b*. Finally, the TTvar was computed by summing all 300 entries of ${\bar{d}}_{E_{}}$. TTvar was computed for all 378 trials.

To evaluate the distribution of variability over the course of the sway trajectory, the trajectory was first split in two phases; the ‘sway endpoint phases’ and the ‘sway traveling phases’, corresponding with the turning points, i.e. outer left and outer right part of the sway, and the part between the turning points respectively. The sway endpoint- and traveling phases consisted of 14 and 16 nodes respectively. The variability per BMU was computed and displayed in Fig 1 as blue ellipses. Secondly the horizontal and vertical component of the variability were computed, as represented by the vertical and horizontal radius of the ellipses. The area representing the variability of the nodes as well as the vertical and horizontal components of the variability, were then averaged for both sway phases, thereby enabling evaluation of phase-related differences in variability.

### Statistical analyses

Statistical analyses were performed using SPSS 20. First, age- and condition effects on sway amplitudes and sway frequency were evaluated using Repeated Measure Analysis of Variance Analyses (RM ANOVA). When main effects of age and condition were observed, a post-hoc analysis using a Bonferroni correction was applied. Secondly, the age- and condition effects on TTvar scores were evaluated. Because assumptions for normality were not met, as assessed by a Shapiro-Wilk test [34], differences between young and older adults were non-parametrically evaluated using a Mann-Whitney U test [35]. Third, the age-, phase- and condition effects on the area and horizontal and vertical component of the variability per node were evaluated, again using RM ANOVA with a Bonferroni correction.

### Classification of BMU trajectories

The second aim of the current study was to train a classifier that allows for discriminating between postural control patterns of young and older adults, based on the SOM features. These features are here defined as the sequences of consecutive BMUs shown as trajectories on the SOM, which can be considered a representation of the movement coordination displayed by subjects during gameplay. To study the possibility of classifying subjects as either young or older adult, based on the BMU-trajectories on the SOM, these trajectories were used to train a second SOM. For each exergame trial, the x and y coordinates of each BMU on the first SOM were stored in a vector ***s*** with dimensions (2 *J* x 1):

$$\text{s}=\left(\begin{array}{c} BMU1_{x}\\ \vdots \\ BMU300_{x}\\ BMU1_{y}\\ \vdots \\ BMU300_{y} \end{array}\right)$$ (11)

The 600-dimensional vectors ***s*** of the trials where the groups differed in TTvar were organized in a matrix **S** with dimensions (*I* x 600), which was used as the input matrix for the second SOM. The output layer again consisted of 625 output nodes with a dimensionality of 600, organized in a (25x25) lattice. In this SOM the BMUs on the output layer can be considered representations of movement coordination of the young and older adults. A k-nearest neighbor (kNN) algorithm [36] was used to identify clusters of these representations of movement coordination of young and older adults on the second SOM. The number of nearest neighbors, k, was set to 5, 70% of the data were used for training the classifier, while 30% was used for testing. Testing was performed 100 times where samples for training and testing were randomly selected in each run.

## Results

### Sway amplitudes and dominant sway frequencies

**Fig 2:**
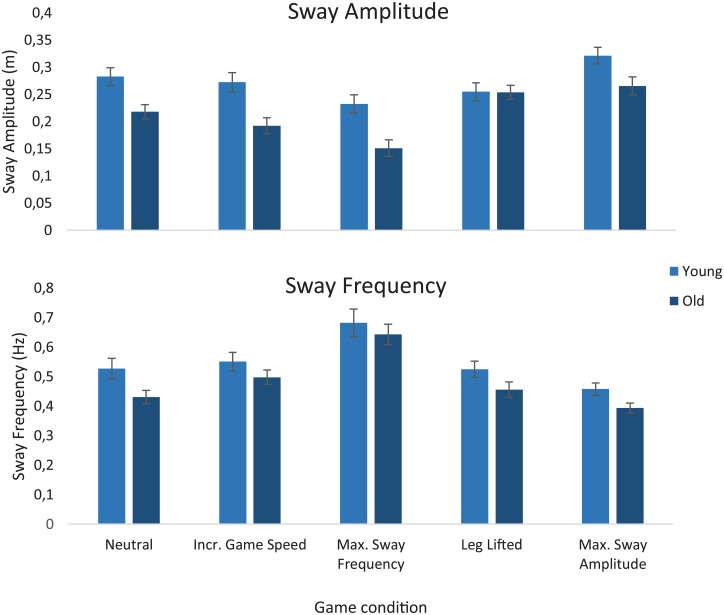
Sway amplitudes and frequencies under different game conditions. Sway amplitudes (top panel) and sway frequencies (lower panel) of young (light bars) and old (dark bars) adults in each game condition. Error bars indicate 1 SEM.

Fig 2 displays the sway amplitudes and sway frequencies of young and older adults under the five task conditions. RM ANOVA showed significant main effects of age and condition on sway amplitude and sway frequency (p<0.01). An age*condition interaction effect was observed for sway amplitude (p<0.01), but not for sway frequency. Post-hoc analysis revealed significant age effects on the conditions ‘maximum sway frequency’ and ‘maximum sway amplitude’ (p<0.01) for both measures, indicating that sway frequency and amplitude were indeed adjusted as a result of these game conditions.

### Movement variability

**Fig 3:**
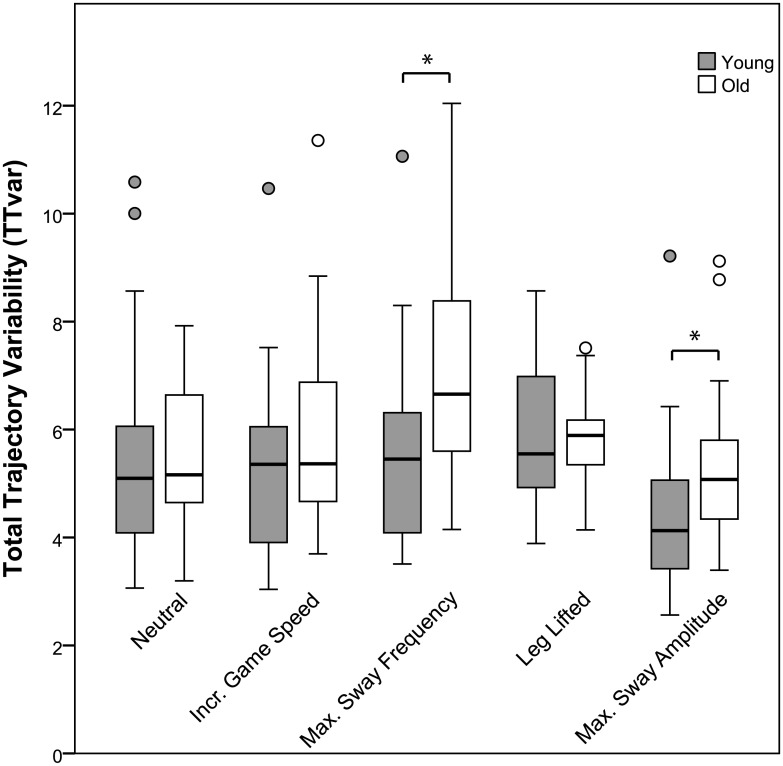
Total trajectory variability (TTvar) of young and older adults in exergame tasks of varying complexity. Total trajectory variability (TTvar) of young and older adults in the five different exergame tasks are displayed by the grey and white boxes, respectively. The line inside the box indicates the median of the sample while the first and third quartile are indicated by the lower and upper part of the box. The error bars represent the size of the range of the total sample, with the exception of outliers, which are indicated with a circle, and defined as samples that are further away from any end of the box than 1.5 times the interquartile range. Tasks on which the groups differ significantly are indicated with an asterisk. Note that TTvar is a measure of distance related to the nodes on the SOM, hence TTvar has no units.

**Fig 4:**
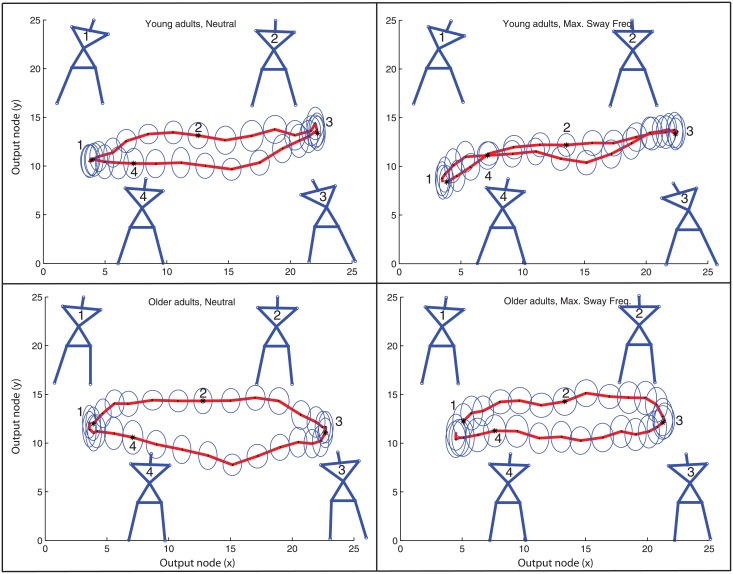
Average BMU trajectories of young and older adults in ‘neutral’ and ‘maximum sway frequency’ conditions. Average sway trajectories displayed by connecting BMUs of subsequent input posture vectors of all young and older adults (upper and lower panels, respectively) in the neutral game condition and maximum sway frequency condition (left and right panels respectively). Blue circles indicate standard errors of the mean of BMU positions. Stick figures indicate the average adopted postures 1, 2, 3 and 4 captured at 1%, 25%, 50% and 80% of the time of the sway movement, respectively.

**Fig 5:**
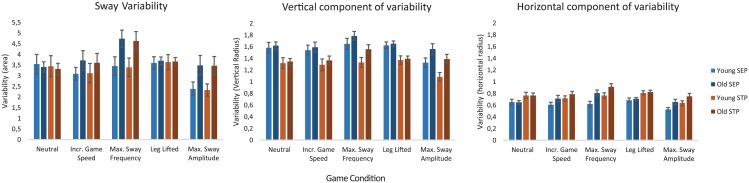
Sway variability and its vertical and horizontal components in different sway phases. Blue and red bars indicate the variability in the Sway Endpoint Phases (SEP), and Sway Traveling Phase (STP) respectively. Light and dark bars indicate young and older adults respectively. Error bars indicate 1 SEM. Note that the variability is a measure of distance related to the nodes on the SOM, hence variability and its vertical and horizontal components are unitless.

Comparison of postural control patterns of young and older adults under various task complexity conditions showed *s*ignificant effects of group on TTvar for the exergame trials where subjects were instructed to sway at maximum sway frequency and maximum sway amplitude (Fig 3). Older adults displayed higher values for TTvar than young adults in these two conditions (7.0 vs 5.5, p = 0.01 and 5.6 vs 4.4, p = 0.02 resp.), indicating that the sway cycles performed by the older adults are more variable than those of young adults, as illustrated in Fig 4. No significant group effects were found for the conditions ‘Neutral’ (p = 0.66), ‘Increased game speed’ (p = 0.31), and ‘Leg lifted’ (p = 0.53). Evaluation of the distribution of variability over the two phases of the trajectory showed a condition effect (p<0.01) on the variability per BMU, but no main effects of age or sway phase. When the variability was split in a vertical and horizontal component (Fig 5), an effect of condition and sway phase on both the vertical and the horizontal component of the variability was observed (p<0.01).

### Classification of BMU trajectories

**Fig 6:**
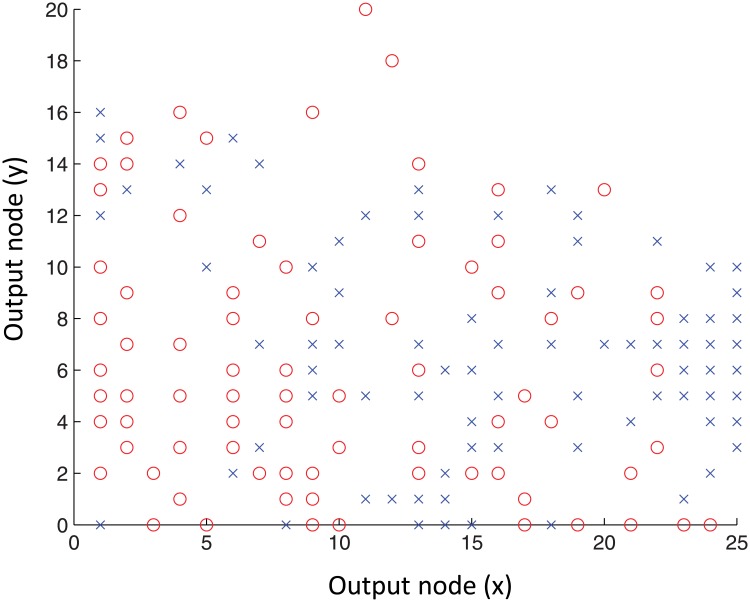
Self-organizing map, trained using the movement patterns of participants under complex exergaming task conditions. Self-organizing map (SOM) trained using the x and y coordinates of consecutive best matching units (BMU) on the first SOM for the trials where young and older adults differed significantly in TTvar. Trajectories of trials of young and older adults were presented to the trained SOM as a single input vector containing all ten sways performed in that trial. The BMU’s matching the trajectories of young (blue x) and older adults (red o) are not spread homogeneously on the map; the lower left corner constitutes of predominantly older adults, whereas many of the trials performed by young adults are organized on the right side of the map.

The BMU-trajectories of young and older adults of the trials where the groups differed significantly in TTvar, which were used for training the second SOM, are not spread homogeneously on the lattice, as shown in Fig 6. Moreover, trials performed by young and older adults appear in clusters connected by regions consisting of a mixture of BMUs associated with young and older adults. The kNN classifier correctly classified 65.8% of the samples.

## Discussion

The main aim of the current study was to examine the differential effects of age and task complexity on movement coordination patterns displayed while the subject is controlling an exergame using weight-shifts. A second aim was to train a classifier that allows for discrimination between young and older adults based on these expressed movement coordination patterns. In the present study, movement patterns and the variability thereof were identified from whole-body movement data, captured during exergaming, using a feature extraction method, i.e. self-organizing maps (SOM). SOM was applied because of its ability to learn underlying non-linear patterns in high-dimensional datasets, while compressing and organizing the information to a low-dimensional visual representation.

In general, analysis of sway characteristics first confirmed that young adults adopted larger and faster sway movements than older adults and that the game conditions had an effect on sway behavior. Secondly, the results showed that older adults display more variability in their movement coordination patterns than young adults under complex task conditions, i.e. in the tasks that required participants to adopt maximum sway frequency and maximum sway amplitude. In the other tasks, where participants performed sway movements at their own comfortable sway speed, i.e. ‘neutral’, ‘increased in-game skating speed’, and ‘leg lifted during sway’, no significant age effects were found in terms of the variability of the identified movement pattern. In the subsequent analyses, a kNN classifier was trained using the movement patterns expressed by the participants during the ‘maximum sway frequency’ and ‘maximum sway amplitude’ conditions, as identified with SOM. The classifier achieved a mediocre accuracy of 65.8% for discriminating between young and older adults.

Our finding that older adults display more variability while performing complex tasks than young adults is consistent with results of previous studies showing that age-related deterioration of postural control is linked with increased movement variability under complex task conditions [16,37,38]. More specifically, the high TTvar score of older adults in the ‘maximum sway frequency’ condition is in line with results of Hernandez et al. and de Vries et al. [37,39] showing that rapid weight shifts are associated with an increased number of submovements as well as decreased fluency and accuracy in the elderly population.

We hypothesized that a trained classifier allows for discriminating between individuals with good and deteriorated postural control, i.e. young and older adults. Although Fig 6 shows that young and older adults seem to cluster on different sides of the map, the classifier achieved an accuracy of only 65.8%, indicating that on an individual level a subject cannot be classified very accurately as either young or old. Fig 6 shows indeed that the age groups seem to behave as a continuum, rather than as clear clusters. This finding could be explained by the broad age range of the young adults; the upper age limit of the young adults was 60, which is close to the lower age limit of the older adults, which was 65 years. Thus, although on an individual level the classification accuracy is only 65.8%, the position of a subject’s movement pattern on the continuum on the SOM holds information about the extent to which the movement pattern expressed during exergaming belongs to the young or to the older group. The accuracy of the kNN classifier could possibly be improved by using more training data. Additionally, the current kNN analysis is only a first step in classification of exergaming movement patterns. Other more advanced classifiers such as Support Vector Machine [30] might further improve the classification accuracy. Moreover, future work should aim at validating the position of movement patterns on the SOM using validated clinical balance measures, thereby enabling more reliable quantification of balance control during exergaming.

Quantification of balance control at short time scales, as endeavored in the current study, opens the door for adjusting the exergame content to the individual users, while they are playing the exergame. Performance-based feedback can then be consistently and systematically altered to create the most appropriate and personalized learning game environment [40]. This principle is generally referred to as Dynamic Difficulty Adjustment (DDA) and is known to facilitate learning and improve motivation to continue playing [41]. Although providing feedback while training motor function is known to improve learning rate and training adherence [42,43], for exergaming positive effects of feedback on motivation and functional outcome have only been reported in a limited number of studies [44–46] and type and frequency of feedback needs to be assessed in future studies. Feedback could also be provided over longer time scales. For instance, improvement of balance ability over days or weeks can be monitored and compared with that of peers, which allows creating competition and setting personal goals, thereby again improving motivation. These extensive monitoring and feedback possibilities contribute to the notion that exergames are particularly suitable for training balance ability in the home environment.

We trained a SOM using 40 participants performing five different task conditions to test for age and task effects. A consequence of the relatively large number of participants and conditions is that a large number of unique poses are captured, which all have to be assigned to nodes on the SOM. As a result, the space on the map occupied by the average sway trajectories seems relatively small (Fig 3), thus these trajectories become coarse. Consequently, subtle differences in TTvar might remain hidden. The current study also showed that the distribution in vertical and horizontal components of variability in sway phases was not equal for the sway phases. The vertical component of the variability is larger in the endpoint phase of the sway, whereas the horizontal component is larger in the traveling phase of the sway. The higher vertical component of variability in the endpoints phase of the sway could be explained by the broader variety in postures that are adopted when getting closer to the limits of stability. The higher horizontal component could be explained by the relatively short amount of time that is spent in the traveling phase of the sway, which results in a coarser representation of the sway trajectory in these phases. A relatively small temporal difference in sway timing thus already leads to a relatively large shift on the map in horizontal direction. Increasing the number of output nodes on the map would provide more detailed information about movement variability. This however, would considerably increase computing time, especially when increasing the amount of data used for training. Since we aimed to develop a balance quantification algorithm for whole-body movement during exergaming, more computing time poses a limitation to the time scale over which the output can be calculated, which has an effect on the time scale on which feedback can be provided.

It should be noted that more space on the SOM is assigned to poses more frequently adopted, at the expense of uncommon poses. Consequently, tasks that require uncommon poses occupy relatively little space on the map, which might result in relatively low values for TTvar, due to the low number of BMUs that define the trajectory. The property of SOM that it can zoom in on frequently adopted poses can be considered a drawback in a research setting aiming at quantification of task effects, but in a clinical setting it is an important advantage. When training a SOM with movement data of large numbers of older adults, it is beneficial to assign little space to very uncommon poses, because many different people can display many different uncommon poses, which would then occupy a lot of space on the map. SOM reduces the impact of uncommon poses, thereby leaving more space on the map for the more regularly adopted poses, which understandably results in a higher resolution (number of nodes) of the BMU trajectories that do not include uncommon poses. When a trajectory occupies more nodes, the subtle changes of this trajectory over time (caused for instance by an improved balance ability of the user) can be observed in more detail, which thus provides more information about changes in movement coordination displayed by the user over time.

In the current study we identified movement patterns and variability therein using SOM. There is however a large body of literature describing other types of computational pattern recognition, including support vector machine [30], deep learning [47], extreme learning machines [48] and many others. We opted for SOM, an unsupervised learning algorithm, because of its ability to structure unlabeled data and because the method is well-evaluated. SOM however holds the downside that it cannot be directly implemented for online monitoring of balance during exergaming. Further development of these pattern recognition algorithms is therefore needed to utilize the full potential of exergames, which can be found in dynamic difficulty adjustment and providing targeted feedback to the user. At that stage of development, exergames might enable older adults to train balance at home, while the system evaluates their balance ability and adjusts the training accordingly by changing difficulty and providing feedback, thereby optimizing the training program on an individual level.

## Conclusions

In this study high dimensional whole-body movement data captured during exergaming were used to identify movement coordination patterns and therewith quantify balance control and train a kNN classifier, able to discriminate between young and older adults. It was found that older adults display more variability than young adults in movement coordination patterns, expressed while performing complex exergame tasks, and that the kNN classifier allows for discriminating between young and older adults based on the movement patterns identified. Our findings contribute to the development of algorithms for quantification of balance ability of older adults during home-based exergaming for balance training. Further development of these algorithms unlock valuable possibilities for adapting exergames to the balance capabilities of individual older adults.

## Supporting Information

S1 Dataset Dataset containing TTvar values of individual young and older participants in all conditions. (XLSX)
